# LiCl Photodissociation on Graphene: A Photochemical Approach to Lithium Intercalation

**DOI:** 10.1021/acsami.1c11654

**Published:** 2021-08-25

**Authors:** Jon Azpeitia, Pablo Merino, Sandra Ruiz-Gómez, Michael Foerster, Lucía Aballe, Mar García-Hernández, José Ángel Martín-Gago, Irene Palacio

**Affiliations:** † Materials Science Factory, Dept. Surfaces, Coatings and Molecular Astrophysics, Institute of Material Science of Madrid (ICMM-CSIC), C/Sor Juana Inés de la Cruz 3, 28049 Madrid, Spain; ‡ Instituto de Física Fundamental, CSIC, Serrano 121, E28006 Madrid, Spain; § ALBA Synchrotron, Carrer de la llum 2-26, Cerdanyola del Vallès, Barcelona 08290, Spain

**Keywords:** lithium, graphene, intercalation, photodissociation, lithium-ion chemistry

## Abstract

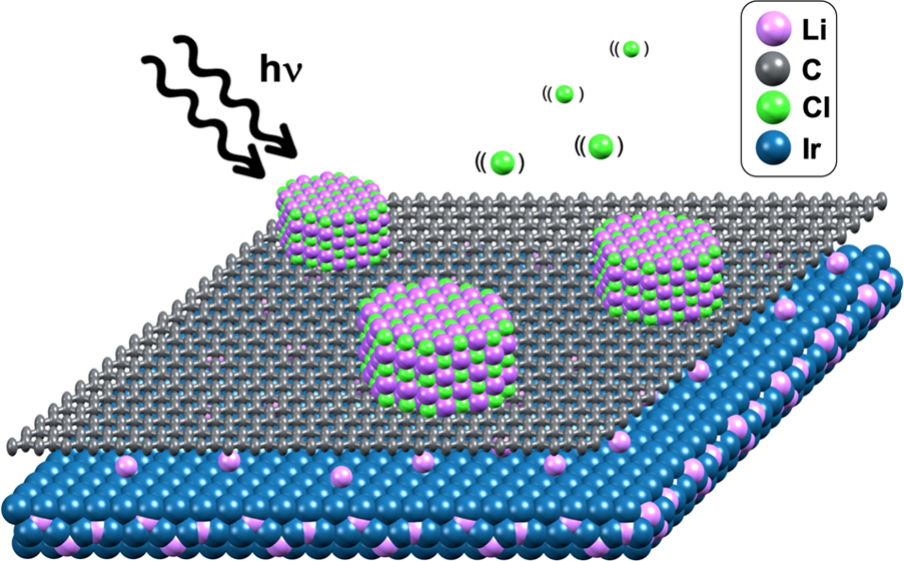

The interest in the research of the structural and electronic properties between graphene and lithium has bloomed since it has been proven that the use of graphene as an anode material in lithium-ion batteries ameliorates their performance and stability. Here, we investigate an alternative route to intercalate lithium underneath epitaxially grown graphene on iridium by means of photon irradiation. We grow thin films of LiCl on top of graphene on Ir(111) and irradiate the system with soft X-ray photons, which leads to a cascade of physicochemical reactions. Upon LiCl photodissociation, we find fast chlorine desorption and a complex sequence of lithium intercalation processes. First, it intercalates, forming a disordered structure between graphene and iridium. On increasing the irradiation time, an ordered Li(1 × 1) surface structure forms, which evolves upon extensive photon irradiation. For sufficiently long exposure times, lithium diffusion within the metal substrate is observed. Thermal annealing allows for efficient lithium desorption and full recovery of the pristine G/Ir(111) system. We follow in detail the photochemical processes using a multitechnique approach, which allows us to correlate the structural, chemical, and electronic properties for every step of the intercalation process of lithium underneath graphene.

## 1 Introduction

Graphene (Gr) has been extensively studied over the past decade,^1^ and there is a significant amount of works regarding the intercalation of small molecules or atoms in graphitic-like nanostructures.^2−4^ In particular, the intercalation of alkali metals under epitaxial graphene has been widely studied^5−7^ since it is a possible route to tailor the graphene band structure and doping level. Among alkali metals, lithium is especially interesting since, in addition to its capability to engineer graphene doping,^8−10^ it bears direct technological applications for graphene-based lithium-ion batteries.^11−14^ In that context, several strategies have appeared focusing on obtaining lithium-intercalated graphene as an anode material by chemical methodologies.^11^ Nanomaterials, and particularly crystalline carbon materials, such as graphite or graphene, play a critical role since they present high mechanical flexibility (volume expansion) and higher electrical conductivity (good electrical contact for several cycles)^15−17^ and enhance the reaction/protection of the active material in batteries.^18^ Moreover, the core processes happening in a lithium-ion battery (charge and discharge) are based on the rocking-chair^19^ or intercalation chemistry. Therefore, alternative routes allowing the intercalation of lithium underneath graphene as well as a detailed description at the atomic level of the structures formed during the intercalation processes may be of interest in the field.

In this work, we present a combined synchrotron-based X-ray photoemission electron microscopy/low-energy electron microscopy (XPEEM/LEEM) study of the Li/Gr system. The combined use of diffracted low-energy electrons and photoelectrons allows us to follow in real time the chemical, structural, and electronic processes occurring during lithium intercalation through graphene. We show that LiCl thin films on top of a single layer of graphene grown on an Ir(111) substrate photodissociate upon exposure to soft X-ray photons. We observe rapid chlorine-ion desorption and a sequence of rapidly evolving lithium intercalation processes. In the first stage, Li intercalates, forming an amorphous two-dimensional (2D) structure that successfully decouples graphene from the Ir(111) substrate. For increasing irradiation time, Li reorganizes below graphene, forming an intercalated (1 × 1) structure. Upon further extensive irradiation, lithium diffuses into subsurface positions in the metal.

## 2 Experimental Methods

All of the experiments have been carried out in the LEEM/PEEM experimental end station of the CIRCE beamline at the ALBA Synchrotron.^20^ Since the sample surface is homogeneous, we generally used the largest available aperture size of 10 μm diameter for microspot measurements, having only occasionally employed the 5 μm diameter one. For repeated or different measurements, the sample was moved to obtain a fresh area for each exposure cycle. Samples were prepared in an ultrahigh vacuum (UHV) chamber with a base pressure of 1 × 10^–10^ mbar. LiCl (≥99.98%, Sigma-Aldrich) was sublimated from a homemade Ta crucible annealed at 720 K controlled by a type-K thermocouple spot-welded to it with the sample kept at room temperature. Ir(111) surfaces were cleaned by repeated cycles of argon-ion sputtering and annealing in an oxygen atmosphere (*T* = 1373 K and *P*_oxygen_= 2 × 10^–8^ mbar). To avoid any residual oxygen on the surface, the last cleaning cycle was carried out without oxygen. Graphene was grown on a single crystal of Ir(111), crystallographic plane (111), in a decomposition process of ethylene. In the first step, ethylene (*P* = 1 × 10^–8^ mbar during 30 s) is adsorbed on the sample at room temperature (RT), and then the sample is flashed up to 1373 K for 30 s. In the second step, the sample is exposed to a higher ethylene pressure (*P* = 1 × 10^–7^ mbar), again followed by thermal decomposition at 1373 K for 7 min.^21^

## 3 Results and Discussion

**Figure 1:**
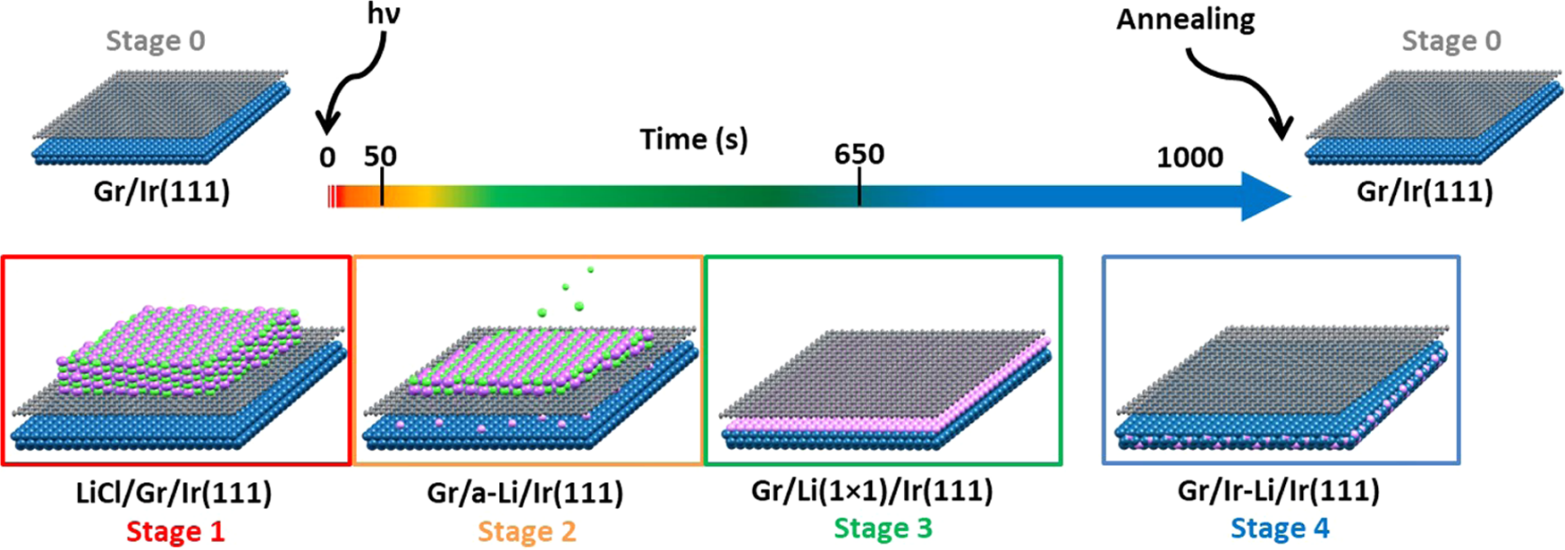
Scheme of the different stages involved in the Li intercalation processes through graphene by photodissociation. Stage 0 represents a pristine Gr/Ir(111) sample. Stage 1 consists of a LiCl thin film grown on top of the graphene. From stage 2 to stage 4, the sample is continuously irradiated with photons (*h*ν = 136 eV). Upon irradiation, LiCl dissociates, Cl desorbs, and Li intercalates through graphene (stage 2). When a full monolayer coverage is reached, Li forms a (1 × 1) structure (stage 3). For longer exposures, Li intercalates deeper into the subsurface region of the Ir(111) (stage 4). Upon annealing, the pristine sample is recovered undamaged (stage 0). The different stages are color coded according to the timeline: stage 1, red; stage 2, orange; stage 3, green; and stage 4, blue. The color of atoms in the scheme is such that purple, green, gray, and blue represent Li, Cl, C, and Ir, respectively.

Figure 1 presents a scheme of the different stages of our experiments. As a starting point, we have a pristine Gr/Ir(111) sample (stage 0). Stage 1 consists of a thin LiCl film (typically 5–7 monolayers thick) grown on top of graphene: LiCl/Gr/Ir(111). The exposition of this system to photon irradiation (*h*ν = 136 eV, photon flux about 2.5 × 10^9^ photons/s·μm^2^) activates the Li photochemistry and triggers the subsequent Li intercalation stages. Immediately after starting the irradiation, the LiCl layers start to photodissociate. During this photo-induced reaction, LiCl dissociates, lithium reduces and intercalates, and chlorine oxidizes, presumably desorbing as Cl_2_, although we cannot rule out the possibility of chlorine-ion desorption without recombination. This leads to stage 2, which is obtained during the first 10–20 s of photon exposure and consists of an amorphous Li layer intercalated between graphene and Ir(111): Gr/a-Li/Ir(111). Because LiCl films are several monolayers thick, there is a gradual process of continuous intercalation that lasts for the first 100 s. When the coverage of intercalated Li reaches the monolayer (ML), lithium orders in a Li(1 × 1) superstructure, resulting in stage 3: Gr/Li(1 × 1)/Ir(111). After long irradiation times, 500–600 s, the LiCl thin film is completely photodissociated, and the next stage of evolution is reached. In stage 4, Li atoms penetrate into Ir(111) first few layers and adopt subsurface positions: Gr/Ir-Li/Ir(111). Stage 4 is the final stable stage observed in our experimental sessions for sufficiently long photon exposure times. It is therefore presumably the most energetically favorable configuration while irradiating. In addition, we note that the pristine G/Ir(111) (stage 0) can be recovered from stage 4 by annealing the sample up to 900 °C.

The full sequence of processes has been studied in real time by a multitechnique in situ characterization in the PEEM/LEEM experimental station at the Alba Synchrotron.^20^ The structure evolution was followed by low-energy electron diffraction (LEED), the chemical evolution was tracked by X-ray photoemission spectroscopy (XPS), and the band structure was measured with microspot angle-resolved photoemission spectroscopy (μ-ARPES). Although we have used an ideal substrate to depict the process, the results can be extrapolated to other sp^2^-based materials, like graphite, graphene on SiC, or free-standing graphene flakes, and more likely to any other 2D van der Waals system.

### 3.1 Structural Evolution

**Figure 2:**
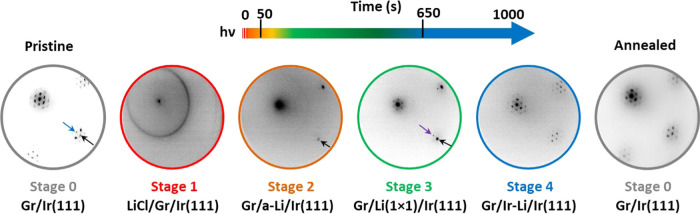
Structural evolution over time and key stages, numbered 0–4, of the irradiated system. Low-energy electron diffraction (LEED) patterns during the photodissociation process (*h*ν = 136 eV) and posterior interaction of Li of the LiCl/Gr/Ir(111) system are presented, and the characteristic irradiation times are represented in the upper arrow. All patterns were acquired with an electron energy of 65 eV. Time 0 s represents the beginning of the irradiation. Black, blue, and purple arrows indicate Gr spots, Ir spots, and Li(1 × 1) spots, respectively.

Figure 2 presents the structural evolution of the system as followed by microspot LEED (see also Supporting Information Movie 1). All of the diffraction patterns were measured at the same electron energy (65 eV) in real time on the same sample, allowing a direct comparison between the different irradiation stages. The largest illumination aperture (10 μm diameter) was used in all cases. Control experiments show that the low-energy electrons of the LEED alone have a negligible effect on the chemistry of LiCl films.

The diffraction pattern of stage 0 is a flower-pattern structure arising from the moiré pattern observed on the pristine Gr/Ir(111) (Figure 2 gray circle).^22^ In this pattern, iridium (blue arrow), graphene (black arrows), and the moiré spots are well resolved. In stage 1, a thin film of LiCl is grown on the sample at room temperature; as a result, a new feature appears in the LEED pattern in the form of a ring (Figure 2 circled in red). The intensity distribution from (0,0) is consistent with the LiCl lattice parameter, and the appearance of a ring-like feature demonstrates that multiple rotational domains of the LiCl crystallites coexist physisorbed on the graphene layer. Because there are no graphene and/or iridium spots, we can estimate the minimal thickness of the LiCl films to be between five and seven monolayers.

At time (*t*) zero, we start photon irradiation at 16° grazing incidence on the LiCl/Gr/Ir(111) sample. In the first 10–20 s, a dramatic change is observed in the diffraction pattern (Figure 2 circled in orange), corresponding to the appearance of stage 2. At this stage, LiCl starts to photodissociate and, while chlorine desorbs (see XPS results below), lithium atoms begin to intercalate through graphene, most probably via structural defects.^23^ In the LEED pattern, we identify this process as a rapid fainting of the LiCl-related ring and appearance of intense graphene spots (black arrows). Indeed, we observed a similar effect during intercalation of NaCl.^22^ Comparison of the evolution with and without X-ray irradiation demonstrates that the primary cause for LiCl dissociation and thus Li intercalation is the illumination from the synchrotron, although we cannot fully rule out a small effect of the electron gun used for LEED, probably in the form of desorption of a small fraction of LiCl. The detection of an intense graphene diffraction pattern and the absence of any moiré spots are clear signs of intercalation-induced decoupling.^24^ It has been reported that submonolayer lithium intercalation can efficiently decouple graphene from the metal substrate, ironing out its corrugation.^25^ At stage 2, neither the Ir(1 × 1) spots nor any other Li-related features are resolved, pointing out the lack of crystallinity in the Li-intercalated layer.^9^

After the first 20 s, stage 2 quickly evolves into stage 3, where the appearance of the Ir(111) (1 × 1) spots (purple arrow) in the diffraction pattern (Figure 2 circled in green) indicates the formation of a Li(1 × 1) superstructure. The Li(1 × 1)/Ir(111) reconstruction after full ML coverage has been reported by Pervan et al.^9^ When Gr/Li(1 × 1)/Ir(111) is reached, the graphene layer remains structurally decoupled from the metal surface and there are no moiré spots in the diffraction pattern. In our experimental sessions, stage 3 lasts over 600 s of photon irradiation time. During this long irradiation time, the background intensity around the (0,0) spot gradually increases and finally the moiré LEED spots start to become visible. A sudden change at around 700 s leads to stage 4 (Figure 2 circled in blue). At this point, as will be shown below, Li stays intercalated under graphene but in addition diffuses into subsurface positions of the iridium substrate. This results in large portions of the metal surface being clean of Li atoms, thus forming a regular layer of Ir(111) just below graphene and hence allowing graphene to couple to Ir, form the moiré structure, and recover the intrinsic Gr/Ir(111) corrugation. This is demonstrated by the reappearance of the moiré spots (Gr/Ir-Li/Ir(111)). Finally, the photon flux is turned off and the sample is annealed up to 900 °C, which leads to a recovered pristine Gr/Ir(111) sample, i.e., stage 0. A full video of the process tracked by LEED is shown in Supporting Information Movie 1.

### 3.2 Chemical Evolution

**Figure 3:**
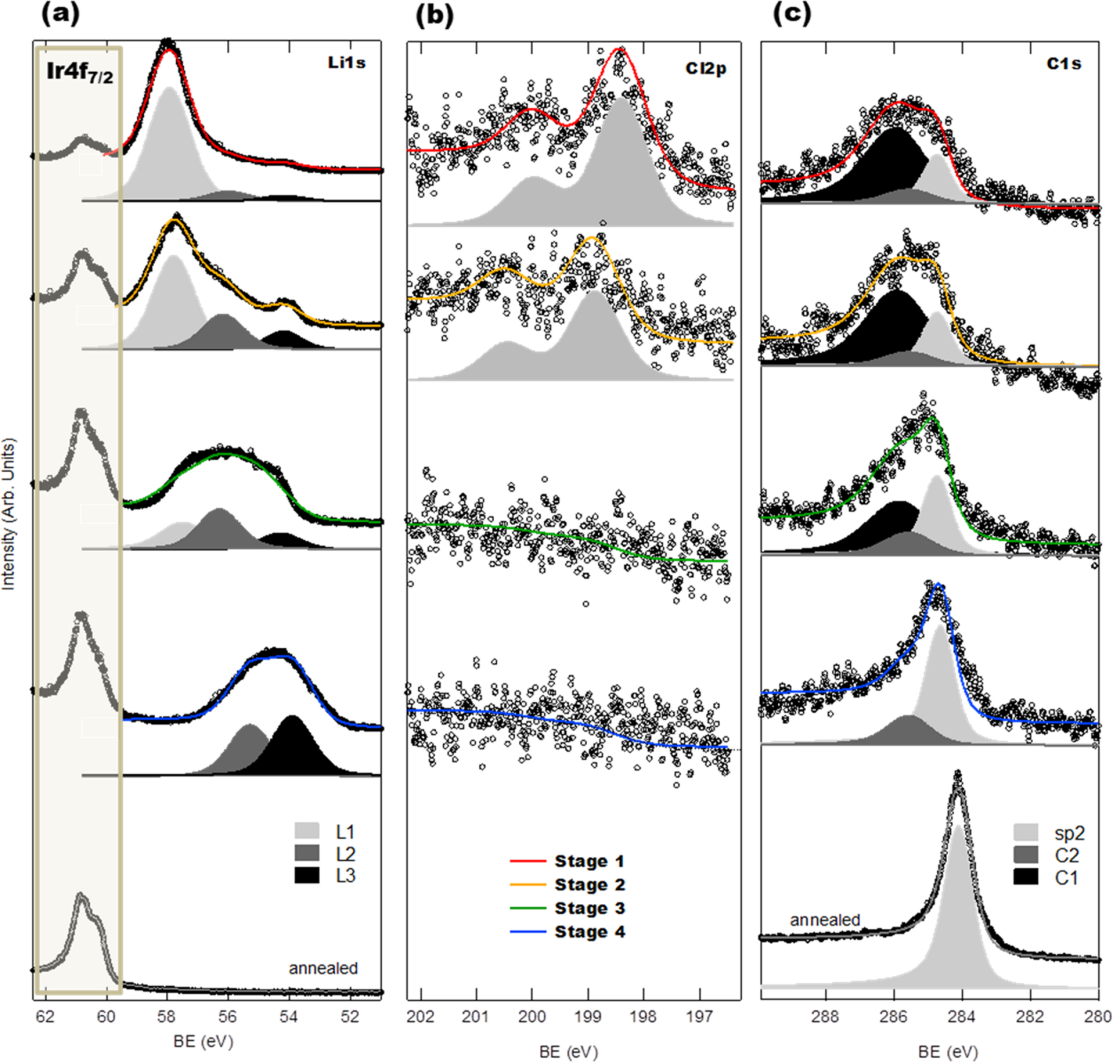
(a) Evolution of the Li 1s core-level spectra and its components of the LiCl/Gr/Ir(111) system upon exposure to photons of 136 eV (red, yellow, blue, and green spectra) and after annealing at 900 °C (gray spectrum). (b, c) Evolution of the Cl 2p and C 1s core levels and their components upon irradiation at *h*ν = 400 eV, respectively.

In the following, we analyze the chemical evolution of the system by tracking the main core levels involved in the process. The Li 1s, Cl 2p, and C 1s XPS peaks were measured using photon energies of 136 eV (Li core-level) and 400 eV (Cl 2p and C 1s core levels), respectively. The photon energy used to measure C 1s and Cl 2p is not the same as the one used for the lithium core-level and for the LEED studies presented above. Control experiments show similar evolution dynamics for both energies, suggesting that the photophysically induced processes are analogous in the X-ray energy range inspected here. Figure 3 summarizes the evolution with exposure time and after annealing at 900 °C of Li 1s (Figure 3a), Cl 2p (Figure 3b), and C 1s (Figure 3c) core-level spectra.

Li 1s and Ir 4f_7/2_ spectra are shown in Figure 3a (see also Figure S1). The Ir 4f_7/2_ core-level appears at a binding energy (BE) of 60.8 eV.^26^ We resolve three different components for the Li 1s (Figure 3a): L1 (at 57.9 eV) is the main component in stage 1 and dramatically decreases upon beam exposure, almost disappearing in stage 3. This component can be assigned to the Li atoms in a LiCl crystal in good agreement with the LEED results (Figure 2). The behavior of L1 matches with the Cl 2p core-level evolution as will be shown below. As L1 starts to decrease, two new peaks at lower BEs emerge, L2 and L3 at 56.1 and 54.3 eV, respectively. We assign L2 to the intercalated lithium sandwiched between the graphene and the iridium surface (stage 2: Gr/a-Li/Ir(111)).^27^ The L2 component intensity increases and shifts toward a lower BE (up to 55.2 eV) as the Li-intercalated coverage increases up to the monolayer saturation coverage and the system reaches stage 3 (Gr/Li(1 × 1)/Ir(111)).^8^ The L3 component also appears in the first stage but dramatically increases in stage 4. We assign this component to Li atoms intercalated below the first iridium layers. At the end of stage 3, LiCl has completely disappeared and the intercalated Li coverage saturates but irradiation continues. As a consequence, Li atoms are forced to intercalate in subsurface positions, and the system would eventually reach stage 4, Gr/Ir-Li/Ir(111).^25^ The presence of the L3 component demonstrates that lithium is present in the sample even though the LEED pattern resembles that of a clean Gr/Ir(111) system. Only after the high-temperature annealing (gray spectrum), all Li 1s components disappear and the structure, chemistry, and energy bands of pristine Gr/Ir(111) are recovered.

If we now turn to the Cl 2p peak^28^ (Figure 3b), we note a strong intensity decrease from the first seconds of irradiation as well as a small shift toward a higher BE. This is a clear indication of chlorine desorption, presumably upon evaporation as Cl_2_, after photodissociation. All traces of the Cl 2p peak disappear during stage 3 in good agreement with the behavior observed for the Li L1 component. Moreover, no other new chlorine-related components are found, indicating that no new chlorine subspecies are formed.

Finally, we focus on the C 1s core-level (Figure 3c). In stage 1, when the LiCl thin film is present on top of graphene and prior to irradiation, C 1s appears as a broad peak with three components. We assign one of them (sp^2^) to C atoms in the sp^2^ configuration and note a small shift (0.7 eV) toward a higher BE with respect to pristine Gr/Ir (284.1 eV),^8,25^ which points toward an effective n-type doping of graphene.^8^ At this stage, there is also a broad component (C1) at a higher BE that we assign to a combination of charge effects as a consequence of the insulating LiCl film and the LiCl/Gr interface (mostly Li atoms interacting with Gr). Component C1 is reduced drastically upon irradiation, disappearing completely at the end of stage 3 where no traces of LiCl can be detected. A smaller component (C2) appearing as a shoulder at a higher BE of the sp^2^ one (0.8 eV w.r.t. sp^2^) remains almost unperturbed during the evolution of the system and is related to the n-doping of graphene. Such a component has been observed in the literature for graphitic systems interacting with lithium and other alkali metals.^8^ After annealing (gray spectrum) and recovering stage 0, the C 1s peak shifts toward a lower BE and narrows, and only the sp^2^ component of graphene on Ir(111) is resolved.^8,25^

### 3.3 Band Structure Evolution

**Figure 4:**
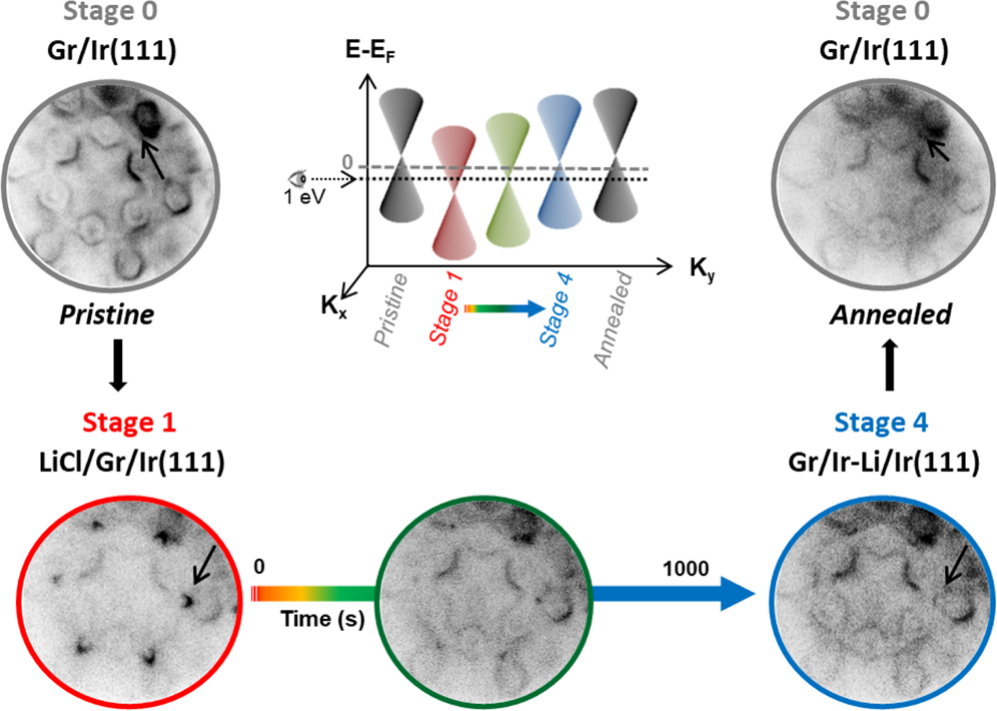
Constant-energy maps at *E* – *E*_F_ = 1 eV of the different stages on photon exposure (*h*ν = 136 eV) from the LiCl/Gr/Ir(111) system to the posterior lithium intercalation. Stage 0 (circled gray) represents the pristine sample Gr/Ir(111), and stages 1–4 represent the stages upon photon exposure. The black arrows point out the changes in one of the dispersive π-bands. Central panel: scheme of the evolution of the Dirac cone through stages 0–4, depicting the p–n–p changes in the doping of the graphene.

All of the structural and chemical changes introduced above will necessarily have a direct impact on the electronic band structure of the system. Using μ-ARPES (*h*ν = 136 eV), we track the changes in the band structure and therefore in the doping of graphene across all of the stages of evolution (see Figure S2 and Supporting Information Movie 2). Figure 4 shows the constant-energy maps at 1 eV below the Fermi level (FL) in stages 0–4, measured in real time under irradiation. The iridium bulk bands and the dispersive π-bands of graphene (indicated by black arrows in Figure 4) at the corners of the hexagonal Brillouin zone, which are the most affected by the photodissociation of LiCl, are simultaneously resolved.

In stage 0, the μ-ARPES energy cuts reveal the p-doped nature of the Gr/Ir(111) system.^29,30^ The growth of a LiCl film on top of graphene leads to a radical displacement of the Dirac cones, resulting in an effective n-doping of the graphene layer (also observed in the XPS spectra, Figure 3c). We measure a displacement of about 1.6 eV of the Dirac point of graphene with respect to stage 0 (see Supporting Information Figure S3), possibly due to electrostatic effects created by the insulating LiCl film (as pointed out by the C1 component in the C 1s core-level, Figure 3c). We note the absence of any measurable gap opening in good agreement with a situation where the LiCl layers physisorb on graphene keeping the sp^2^ structure intact. When the sample is irradiated, we observe a strong and fast shift of the dispersive π-bands (marked in Figure 4 by black arrows). During this process, the system evolves through stage 1, to stage 2 (Gr/a-Li/Ir(111)), to stage 3 (Gr/Li(1 × 1)/Ir(111)), and ends up in stage 4 (Gr/Ir-Li/Ir(111)). The rapid evolution of the system and the long integration times needed for obtaining low-noise μ-ARPES maps hinder precise measurements of the doping shift in the nonequilibrium stages (stages 2 and 3). However, comparison with the literature allows us to conclude that we obtain the expected n-doping of a Li-intercalated graphene sample of around 1.52^5^–1.63 eV.^9^ In stage 4, the Dirac cones almost recover their original pristine state. This is consistent with the situation where lithium is no longer underneath graphene but in a subsurface position. As expected, the Gr/Ir(111) bands are completely recovered after annealing.

## 4 Conclusions

In summary, we present a method to intercalate lithium at the graphene/iridium interface by photodissociation of an adsorbed LiCl thin film. A combined characterization with LEEM, XPS, and μ-ARPES allows us to follow every step of the intercalation process in real time. We show that upon photon exposure, LiCl dissociates, chlorine atoms desorb, and lithium intercalates graphene. This is evidenced by the changes of the diffraction patterns, the fast decrease of Cl 2p core-level intensity, and the evolution of the Li 1s peak. When the intercalated lithium amounts to one monolayer, a 1 × 1 structure appears in the LEED pattern. Finally, the process reaches a final state with lithium remaining in a subsurface position in the iridium substrate, as evidenced by the lithium core-level signal and the LEED pattern. μ-ARPES measurements show a high n-doped graphene behavior from the beginning of the Li intercalation processes. As the intercalation process takes over, graphene bands evolve to an effective p-doped behavior. The photo-induced sequence does not damage the graphene layer, as evidenced by the fact that the Gr/Ir(111) original stage can be recovered upon high-temperature annealing. Our fundamental study contributes to a better understanding of the Li intercalation process in graphitic materials upon photophysical methods, which may lead to new pathways for the improvement of next-generation graphene-based anodes for lithium-ion batteries.

## Supporting Information Available

Detailed evolution of the L 1s core-level upon photon irradiation (*h*ν = 136 eV) of a LiCl film grown on top of a Gr/Ir(111) substrate; complete set of constant-energy maps at different energies on photon exposure time for the photo-induced Li intercalation in a Gr/Ir(111) sample; constant-energy maps taken at different *E* – *E*_F_ energies (*h*ν = 136 eV) from 0.25 up to 2.6 eV below the Fermi level for stage 1 of the LiCl/Gr/Ir(111) system (PDF); 50 μm field of view Low Energy Electron Microscopy (LEEM) image of a LiCl/Gr/Ir(111) sample; and estimation of the LiCl photodissociation efficiency.
In situ microspot LEED video at 65 eV while the LiCl/Gr/Ir(111) sample is irradiated with photons at 136 eV. The changes that can be appreciated during the video show how the surface structure of the system evolves upon irradiation. The starting diffraction pattern is the one of a LiCl film grown on top of a Gr/Ir(111) sample. The irradiation starts the moment the first changes are observed in the diffraction pattern. The video reveals the fast transformation leading to the photodissociation of LiCl to the posterior intercalation of Li from a structural point of view (Movie 1) (AVI)
In situ μ-ARPES video showing the evolution of the iridium bulk bands and the dispersive π-bands during photon exposure at 136 eV. The video shows a constant-energy map at *E-E*_f_ = 0.85 eV and its evolution during photon irradiation of a LiCl/Gr/Ir(111) sample. As changes occur very fast, to be able to track them, we first start in an already exposed region of the sample, and then we move into a “fresh” zone. The μ-ARPES video reveals the shift of the dispersive π-bands, from a heavily n-doped to a slightly p-doped graphene when the photoinduced process is finished and the remaining lithium settles in a subsurface position in the iridium (Movie 2) (AVI)

## Supplementary Material

am1c11654_si_001.pdf

am1c11654_si_003.avi

am1c11654_si_004.avi
