# Supplementary material for: LiCl Photodissociation on Graphene: A Photochemical Approach to Lithium Intercalation
Source: ACS Appl Mater Interfaces. 2021 Aug 25;13(35):42205–11. doi: 10.1021/acsami.1c11654 (PMC8431332; doi:10.1021/acsami.1c11654)
Supplement: Supplementary file 1 — am1c11654_si_001.pdf [file am1c11654_si_001.pdf]

**SUPPLEMENTARY INFORMATION**

# LiCl photodissociation on graphene: A photochemical approach to lithium intercalation

*Jon Azpeitia[1], Pablo Merino[1,2], Sandra Ruiz-Gómez[3], Michael Foerster [3], Lucía Aballe[3], Mar García-Hernández[1], José Ángel Martín-Gago[1], Irene Palacio [1]**

[1]Materials Science Factory, Dept. Surfaces, Coatings and Molecular Astrophysics, Institute of Material Science of Madrid (ICMM-CSIC), C/Sor Juana Inés de la Cruz 3, 28049 Madrid, Spain.

[2] Instituto de Física Fundamental, CSIC, Serrano 121, E28006, Madrid, Spain.

[3] ALBA Synchrotron, Carrer de la llum 2-26, Cerdanyola del Vallès, Barcelona 08290, Spain.

**Corresponding Author**

* i.palacio@csic.es

## 1. XPS: Evolution of Li1s core level upon long-time photon irradiation

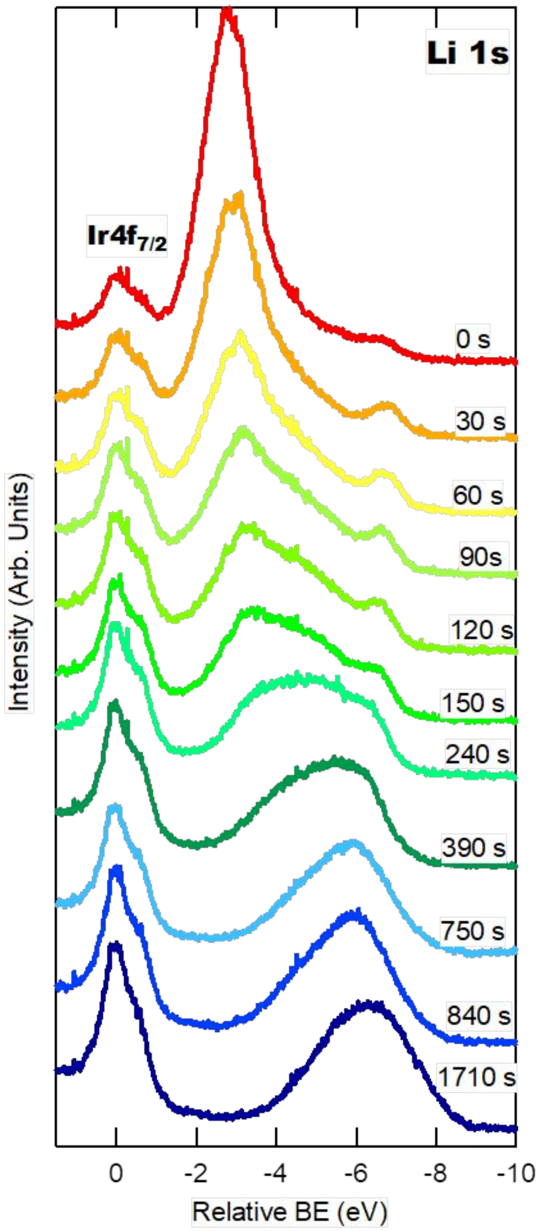


***Figure S1.*** *Detailed evolution of the L1s core level upon photon irradiation (hv=136 eV) during 1710 seconds. The peak of Ir4f$_{7/2}$ (60.8 eV [1]) has been taken as a reference for the binding energy (BE). The starting point sample consists of a LiCl film grown on top of a Gr/Ir(111) substrate. The photon exposure starts at 0 seconds and continues over 1710 s. The red spectrum corresponds to stage 1 of the main text, LiCl/Gr/Ir(111),*

*and the subsequent spectra to stages 2 to 4, where LiCl first dissociates and then intercalates trough graphene to end up in a subsurface position of the iridium substrate.*

## 2. µ-ARPES: Evolution of the dispersive π- bands during the LiCl photodissociation on graphene.

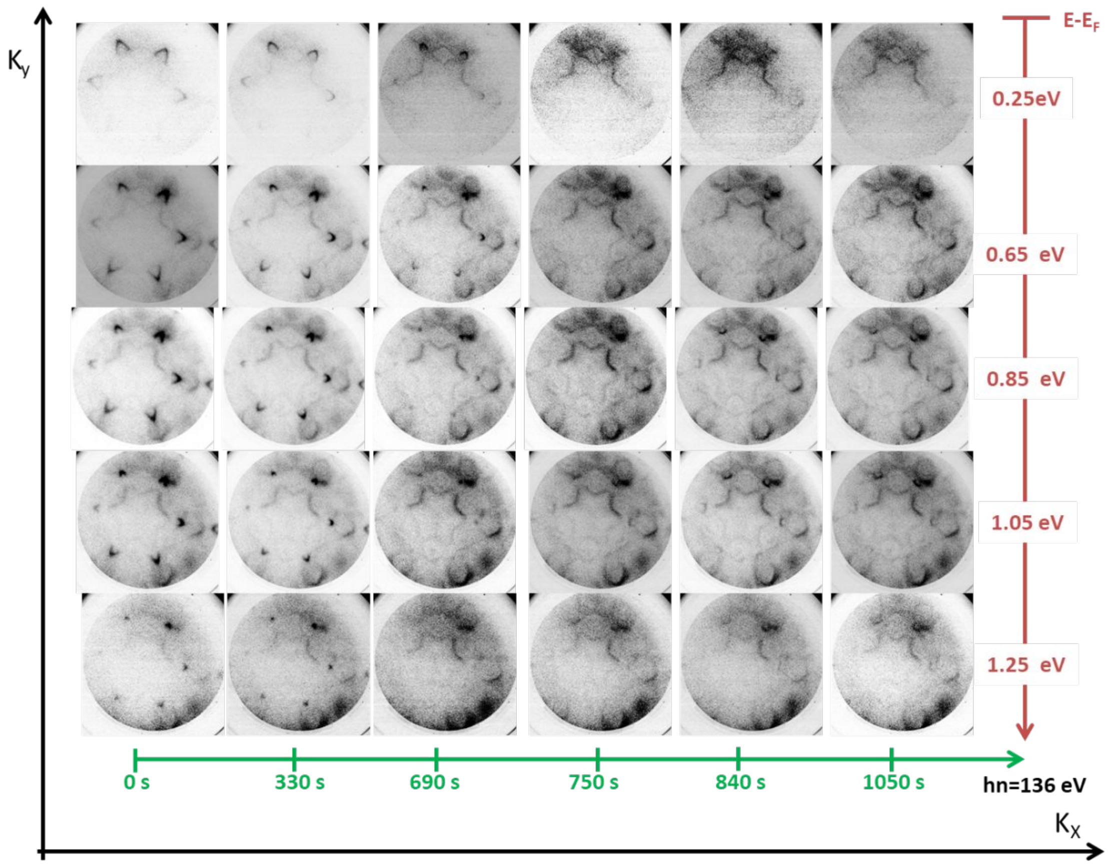


***Figure S2.*** *Complete set of constant energy maps at different energies ($E-E_F$) over photon exposure time for the photo-induced Li intercalation in a Gr/Ir(111) sample. The photon energy used is 136 eV, x-axis (green one) follows the exposure time evolution of the system upon photon irradiation and y-axis (red one) indicates the energy ($E-E_F$) at which the map has been taken from 0.25 eV down to 1.25 eV below Fermi level. Time 0 seconds is defined as stage 1 in the main text, and corresponds to a LiCl film on top a Gr/Ir(111) sample. The continued photon exposure triggers a fast shift of the dispersive π-bands, from a heavily n-doped graphene towards p-doping.*

3. **µ-ARPES: Constant energy maps taken at different E-E$_f$ energies of stage 1: LiCl/Gr/Ir(111).**

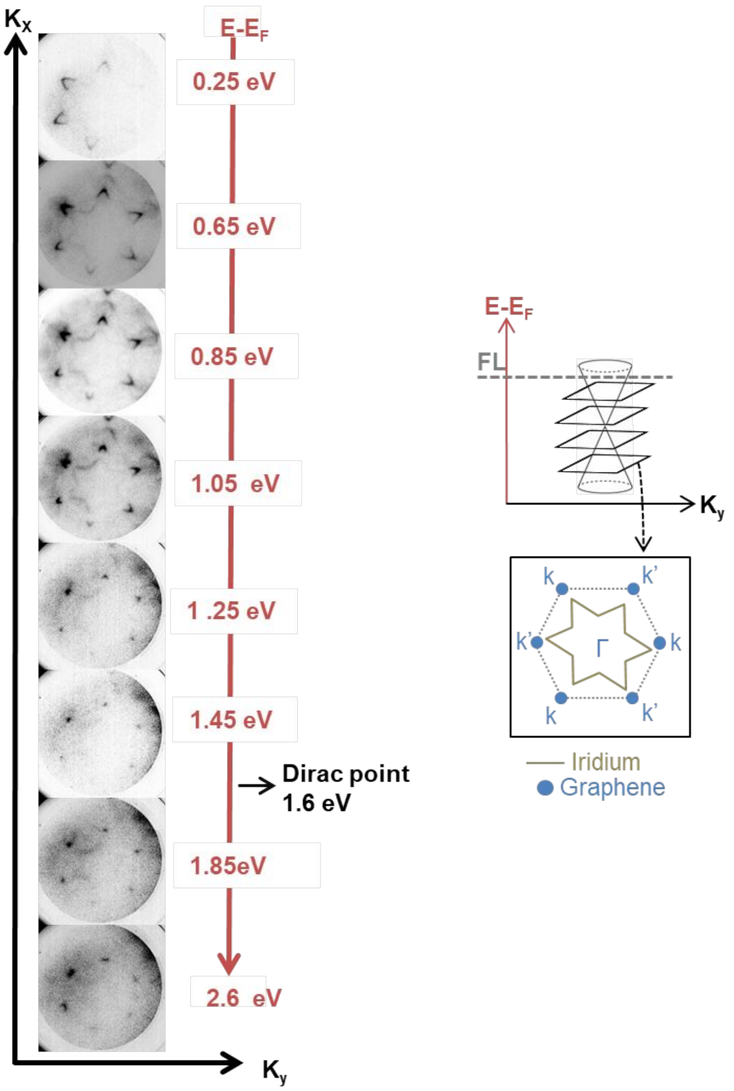


***Figure S3.*** *Constant energy maps taken at different E-E$_f$ energies (hv=136 eV), from 0.25 eV up to 2.6 eV below the Fermi level for the LiCl/Gr/Ir(111) system (stage 1 in the main text). The distinctive Dirac cones are visible at the corners of the hexagonal Brillouin zone as well as the iridium bulk bands (scheme on the right panel as a guide to the eye). A shift of the Dirac point about 1.6 eV below the Fermi Leve is shown.*

4. **LEEM image of a LiCl/Gr/Ir(111) sample partially irradiated**

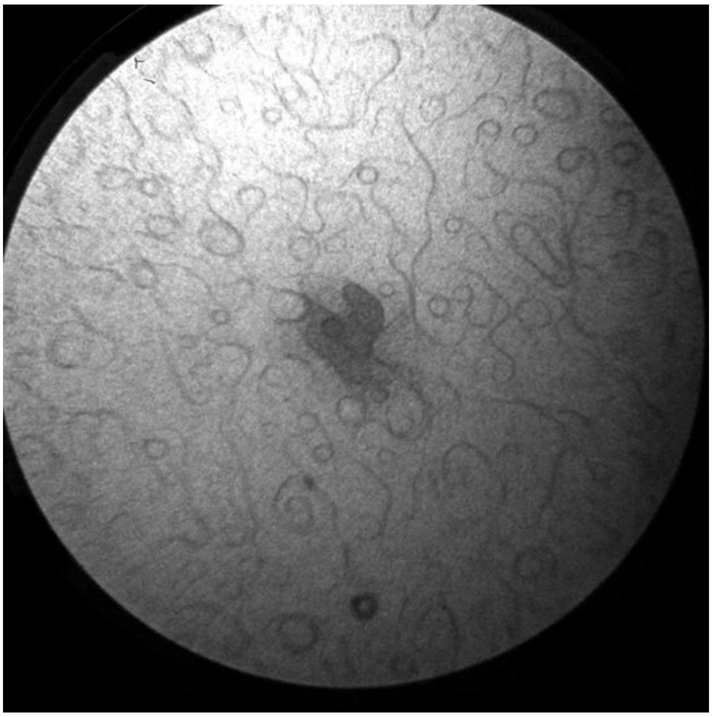

***Figure S4.*** *50 μm field of view Low Energy Electron Microscopy (LEEM) image of a LiCl/Gr/Ir(111) sample. The irradiated area of the sample can be appreciated as a black area in the center of the image. Also numerous Ir atomic steps (thin lines) and wrinkles (thick lines) are visible.*

5. **Estimation of the LiCl photodissociation efficiency**

We have done an estimation of the photodissociation process efficiency assuming that it is proportional to the Li-Cl broken bonds (events) per photon. This could be written as follows:

$$E \sim \frac{\rho \cdot h}{\phi t}$$

where h is the thickness of the LiCl layer ($h = a \cdot l$ , where $a$ is the lattice parameter and $l$ the number of monolayers of LiCl), $\rho$ the number of bonds per

volume ($\rho = \frac{3}{a^3}$), $\Phi$ the photon flux, $t$ the total time needed to break the LiCl bonds.

In our experiment, with a certain configuration set-up and for hν 136 eV, we have estimated a thickness of the LiCl film of about 5-7 monolayers, the flux is about $2.5 \times 10^9$ photons/s·μm$^2$ and the total time needed to break all the LiCl (basically when chlorine is no longer detected) is about 150 s. Hence we obtain an efficiency of about $7\times10^{-4}$ events/photon.

REFERENCES

(1) Grånäs, E.; Knudsen, J.; Schröder, U. A.; Gerber, T.; Busse, C.; Arman, M. A.; Schulte, K.; Andersen, J. N.; Michely, T. Oxygen Intercalation under Graphene on Ir(111): Energetics, Kinetics, and the Role of Graphene Edges. *ACS Nano* **2012**, *6* (11), 9951–9963.
